# Supplementary material for: Are carbon emissions trading and green financial instruments synergistic? -Comprehensive quantitative research based on content analysis
Source: PLoS One. 2024 Mar 7;19(3):e0298601. doi: 10.1371/journal.pone.0298601 (PMC10919601; doi:10.1371/journal.pone.0298601)
Supplement: S1 Data — (ZIP) [file pone.0298601.s001.zip › Data/地级市Z_F工作报告.zip!/╔╜╢1⁄2/╤╠╠¿╩╨/└┤╫╘╣1⁄2╓┌║┼í╛┬φ┐╦╩2╛▌═°í┐_2009.docx]

各位代表：

现在，我代表市人民政府向大会报告工作，请予审议，并请市政协委员和其他列席会议的同志提出意见。

一、2008年政府工作回顾

2008年是极不平凡的一年。面对复杂多变的国际形势和历史罕见的严峻挑战，全市上下在中共烟台市委的坚强领导下，坚持以邓小平理论和“三个代表”重要思想为指导，深入落实科学发展观，认真贯彻党的十七大和十七届三中全会精神，科学把握形势，积极应对挑战，努力克服重重困难，扎实推进各项工作，经济社会实现了平稳较快发展。初步核算，全市生产总值完成3434.2亿元，比上年增长13.6%。境内税收收入达到348.6亿元，地方财政收入达到166.2亿元，分别增长20.1%和18%。固定资产投资完成1953.8亿元，增长24.1%。城市居民人均可支配收入达到19350元，农民人均纯收入达到7935元，分别增长15.4%和13.7%。

——三次产业健康发展，经济增长的协调性、稳定性进一步增强。工业经济实现速度效益同步增长。全市规模以上工业完成增加值2005.7亿元，增长17.5%，利税、利润分别增长17.1%和17%。机械制造、电子信息、食品加工和黄金四大支柱产业完成主营业务收入4577.6亿元，占规模以上工业的比重达到59.4%，石化、核电、冶金、生物制药、葡萄酒等产业发展势头强劲。主营业务收入过10亿元企业达到97家，其中过百亿元企业达到9家，分别增加10家和3家。高新技术产业产值达到3146.5亿元，增长36.9%。新增国家级企业技术中心2家、中国驰名商标9件。服务业实现较快发展。全年完成服务业增加值1067.7亿元，增长16.5%。社会消费品零售额完成1023.4亿元，增长23.4%。港口货物吞吐量达到1.47亿吨，其中集装箱达到153万标箱，分别增长11.8%和22.5%。成功举办了第二届烟台国际葡萄酒节、第十届国际果蔬·食品博览会等各类展会25个。旅游业实现总收入228.5亿元，接待海内外游客2381万人次。服务业“双50”工程进展顺利。国家级动漫产业基地入驻企业达到36家。全市金融机构存贷款余额分别增长19.6%和14.5%，民生银行在我市设立支行。“家电下乡”试点工作取得明显成效。农业综合产出能力持续增强。全面落实各项强农惠农政策，为种粮农民发放粮食直补、农资综合补贴2亿元。粮食生产喜获丰收，全市玉米平均单产首次突破1千斤。肉、蛋、奶产量分别达到40.2万吨、28.5万吨和27.8万吨，水产品产量达到184万吨。“烟台苹果”等4项产品获注地理标志证明商标。国家和省级重点农业龙头企业分别达到11家和46家。

——改革开放不断深化，经济国际化、市场化水平进一步提高。对外贸易保持强势增长。全市完成外贸进出口总额350.3亿美元，其中出口206.5亿美元，分别增长46.3%和46.5%，出口增幅居全国首批14个沿海开放城市之首。出口过亿美元的企业15家，富士康成为全省最大的进出口企业。机电产品和高新技术产品出口比重分别达到66.8%和36.5%。利用外资稳定增长。注册外资额达到16.7亿美元，实际到账外资10.6亿美元，分别增长7.2%和12%。“走出去”战略步伐加快。境外投资协议额达到8000万美元，增长200%。中俄——托木斯克木材工贸合作区建设开始启动。对外承包工程与劳务合作合同额、营业额分别增长19.3%和8.4%。烟台经济技术开发区龙头带动作用持续增强。企业改革成效明显。完成了17家市级国有企业改制任务，新增烟台氨纶、恒邦股份、民和股份3家上市公司。民营经济规模和层次快速提升。新发展个体工商户26510户、私营企业6647家，民营经济实现增加值2600亿元，上缴税金突破200亿元。农村综合改革进一步深化。土地承包经营权流转速度加快，全年土地流转面积达到19.9万亩，占发包总面积的3.6%。财税金融、行政审批制度、市政公用事业、文化卫生体制等各领域改革取得新的进展。

——城乡建设统筹推进，区域发展活力进一步提升。城市规划、建设和管理工作全面加强。一批城建重点项目顺利实施，火车站、文化中心主体工程基本完工，北马路拓宽改造全线恢复通车，大南山生态景区项目建设有序进展，迎春大街和幸福区片综合整治工程、大海阳和毓西区片“平改坡”工程快速推进。新建和延伸了山海南路、通世南路、青年南路、滨海路等15条城市道路，新建和改造了套子湾、辛安河二期等9个污水处理厂，实施了门楼水库上游治理、逛荡河整治和市区雨污分流配套工程，建设整治了20处社区公园。一批能源交通项目取得重大进展，海阳核电一期进入主体工程建设阶段，八角热电、华电国际、百年电力和天然气第二气源等项目前期工作进展顺利；蓝烟铁路电气化改造工程开工建设；烟台港西港区液体化工码头、芝罘湾港区三突堤集装箱码头完成主体工程；206国道蓬莱至龙口、龙口至招远等7条公路建成通车；莱山机场新建主跑道正式启用，烟台潮水国际机场项目列入省重点工程。五区融合步伐加快，基础设施和产业对接更加紧密。城市管理体制进一步理顺，数字化城市管理系统建成运行。新农村建设迈出坚实步伐。组织开展了418个村庄的环境综合整治，清洁治理了814个村庄的河溪池塘，新发展沼气用户8000户，建成农村文化大院3924个，改扩建敬老院113处。改造硬化农村公路639公里，新建改建了680个村庄的自来水工程，农村公路硬化率、自来水普及率分别达到98%和96.1%。完成了1座中型水库和179座小型水库除险加固任务，胶东引黄调水控制性工程全面完工。“三年大造林”工程当年完成造林绿化面积20.1万亩，全市林木覆盖率达到38%。

——社会事业更加繁荣，群众生活质量进一步改善。中科院烟台海岸带研究所一期开始启用，中科院烟台综合技术转化中心正式成立。全市有5个项目列入国家科技支撑计划，完成专利申请5435件，取得市级以上科技成果187项，获国家发明二等奖1项、科技进步二等奖3项，我市入选中国城市综合创新五十强。人才强市战略深入实施，引进各类人才2.6万人。全部免除城乡义务教育阶段学生课本费，素质教育水平不断提高，职业教育和高等教育发展能力持续增强。完善公共文化服务体系，广泛开展纪念改革开放30周年系列宣传活动，群众文化生活日益丰富。加强城乡卫生服务体系建设，城市社区卫生服务机构覆盖率达到94.5%，新农合参合农民补助标准由每年人均60元提高到80元。城乡群众健身条件进一步改善，烟台籍运动员在北京奥运会取得了1金1铜的好成绩，我市获得“振兴山东体育突出贡献奖”。全年新增城镇就业14.3万人，转移农村劳动力3万人，城镇登记失业率控制在3.1%，城镇居民基本医疗保险覆盖率达到68.4%，各类社会保险待遇按时足额发放率保持在100%。提高了城乡低保标准，城市低保每人月均302元，农村低保每人年均1300元，分别提高37元和162元。深入开展爱心捐助活动，全年发放救助金近6000万元，救助困难群众23万余人。全市新建经济适用房、廉租房等保障性住房3756套。2008年度为民服务十件实事全部完成。帮扶栖霞、支持长岛和对口支援工作取得新的成果。人口和计划生育工作继续走在全省前列，气象服务水平进一步提高，老龄、妇女、儿童、红十字和残疾人事业得到加强，广播电视、新闻出版、防震减灾、史志档案、国防动员、双拥共建、人民防空、民族宗教等工作取得新的成绩。

———民主法制继续加强，和谐稳定局面进一步巩固。深入开展创建全国文明城市活动，积极建设和谐稳定模范城市，城乡文明状况和社会治安秩序继续好转。重视和加强信访工作，健全矛盾纠纷排查疏导化解机制，集中解决了一批群众关心的热点难点问题。安全生产形势持续稳定，食品药品监管力度逐步加大。坚持依法行政，健全重大决策专家咨询、社会公示和听证制度，完善科学民主决策机制，全面推行行政执法责任制。各级政府主动接受人大、政协及各民主党派、工商联和社会各界的监督，认真办理人大代表建议和政协提案。加强公务员队伍建设，加大行政监察力度，深入开展行风评议、纠风专项治理和优化发展环境活动，坚决纠正损害群众利益的不正之风。强化廉政建设，严格遵守领导干部廉洁自律规定，认真查处违纪违法案件，进一步树立了政府机关和公务员队伍的良好形象。

各位代表！刚刚过去的2008年，是我们经受考验、奋力开拓的一年。一年来，面对党和人民赋予的历史重任，各级埋头苦干、狠抓落实，有效推进了一批事关全局和长远发展的重大项目和重点工作。新一届政府为适应国内外形势的新变化，适应全市人民过上更好生活的新期待，一心一意谋发展，突出重点抓大事，铺下身子抓落实。坚持不懈地培植大型骨干企业，积极开展项目推进年、开工月、促进月活动，加大对城市经济、海洋经济、临港产业、财源建设等工作的调度推进力度，集中精力解决工作中的困难和问题，培育了一批产业龙头和新的经济增长点，拓展了经济发展的新空间。一年来，面对科学发展提出的更高要求，各级转变观念、创新工作，始终坚持了资源节约型、环境友好型的发展方向。着力转变发展方式，坚持有增有减的原则，努力提高增长性指标，坚决控制约束性指标。在发展任务越来越艰巨、区域竞争越来越激烈的情况下，坚持把节能减排作为一项硬任务，采取刚性措施，关停了26.3万千瓦小火电机组、22条立窑水泥生产线。全市万元生产总值能耗继续下降，预计SO2和COD排放分别下降4.2%和7.3%。一年来，面对前所未有的困难挑战，各级科学运筹、加强调控，努力把各种不利因素影响降到了最低限度。统筹安排、合理调度各类要素资源，有力化解了夏季煤电油运高度紧张的矛盾。多方组织紧缺物资储备和供应，为相关企业和困难群体发放各类补贴5.4亿元，有效缓解了物价上涨的压力。全力应对百年不遇的国际金融危机，迅速启动扩内需、保增长的重点项目，积极争取上级资金支持，鼓励和引导金融机构加大信贷投放，帮助外经贸企业开拓市场、解决困难，出台一系列扶持中小企业、促进房地产业健康发展的政策措施，使全市经济在逆境中实现了平稳较快增长。一年来，面对频繁发生的各类突发事件，各级齐心协力、团结奋战，保持了社会的安全、稳定与和谐。积极参与“4.28”胶济铁路事故、青岛海域浒苔灾害的应急处理，妥善处置了问题奶粉和氯气泄露等重大突发事件，圆满完成了奥运安保任务。四川汶川特大地震灾害发生后，社会各界心系灾区、踊跃援助，前方后方全力以赴、密切配合，取得了抗震救灾的重大阶段性成果，得到了灾区人民的普遍赞誉。我市卫生防疫队被授予“全国抗震救灾英雄集体”称号。截止去年底，全市共捐款捐物4.5亿元，已向灾区划拨2亿多元，妥善救治和安置了68名灾区伤员、120名灾区学生，启动了第一批援助灾区恢复重建项目。这次抗震救灾，是对我市广大干部群众的一次重大考验。灾难面前，全市人民以自己的爱心和实际行动，经受住了考验，展示了不屈不挠、顽强拼搏的民族精神，谱写了患难与共、众志成城的时代篇章！

各位代表！过去的一年，在国际经济环境急转直下、国内困难压力明显加大的情况下，我市经济社会发展所取得的成绩来之不易！这些成绩的取得，是上级党委、政府和中共烟台市委科学决策、正确领导的结果，是市人大、市政协和各民主党派有效监督、大力支持的结果，是社会各界和全市人民万众一心、团结拼搏的结果。在此，我代表市人民政府，向全市广大工人、农民、知识分子、各级干部以及各阶层的建设者，向驻烟人民解放军、武警部队官兵和政法干警，向中央、省属驻烟单位，向各位人大代表和政协委员，向各民主党派、工商联、无党派人士和各社会团体，向所有关心支持烟台发展的港澳同胞、台湾同胞、海外侨胞和国际友人，致以崇高的敬意和衷心的感谢！

各位代表！我市经济社会发展虽然取得了很大成绩，但也存在不少困难和问题，主要表现在：经济结构性矛盾在新形势下表现得更加突出，抗击国际市场风险的能力比较脆弱；发展方式还没有根本转变，自主创新能力和节能减排水平需要进一步提高；企业发展的制约因素增多，工业增幅回落较快；外经贸形势十分严峻，招商引资和外贸出口的增长势头有所减缓；就业压力增大，一些群众关心的热点难点问题解决得还不够好；农民增收、财政增收面临较大困难，经济与社会、城市与农村以及县域之间发展不平衡的问题仍比较突出；发展环境有待进一步优化，政府自身建设还有差距，作风不实、效率不高、为政不廉的现象不同程度地存在，等等。这些问题，我们一定高度重视，认真加以解决。

二、2009年政府工作安排

2009年是新中国成立60周年。做好今年的工作，意义十分重大。当前，国际金融危机快速蔓延，对实体经济的影响正进一步加深。我市作为全国首批14个沿海开放城市，经济外向度高，对国际市场的依赖性强，受到的冲击将尤为严重。保持又好又快的发展态势，任务异常艰巨，我们将面临比以往更加严峻的考验。但越是在复杂多变的形势之下，越是在困难面前，我们越要保持清醒头脑、树立必胜信念。要看到，这次危机是全球性的，所有城市都会面临同样的风险和挑战。能不能转危为机、渡过难关，关键看如何把握、如何调整、如何化解。我们更要看到，我国经济发展的基本面和长期趋势没有改变，主导和支撑中国经济发展的工业化、城市化进程没有放缓，国家采取的一系列扩大内需、促进增长的宏观政策为我们带来了新的机遇和更大的发展空间。经过30年的改革开放和发展积累，我市已经具备了继续保持较快发展的物质基础、智力支撑和干部队伍保障。只要全市上下坚定信心、迎难而上，不为任何风险所惧，不被任何干扰所惑，就一定能够变压力为动力，化挑战为机遇，战胜各种艰难险阻，在前进的道路上取得更大成绩。

今年政府工作的主要任务是，全面贯彻党的十七大和十七届三中全会精神，坚持以邓小平理论和“三个代表”重要思想为指导，深入贯彻落实科学发展观，把保持经济平稳较快发展作为首要任务，解放思想、提升境界，积极作为、科学务实，加快转变发展方式，着力调整经济结构，千方百计扩大投资和启动内需，更加重视深化改革和扩大开放，全面推进自主创新和节能减排，高度关注民生保障和社会建设，努力实现又好又快发展，全面建设文明富庶新烟台。

经济社会发展主要预期目标是：全市生产总值增长11%；地方财政收入增长10%；固定资产投资增长20%；社会消费品零售额增长16%；利用外资在改善结构的基础上保持适当规模，外贸进出口总额增长6.5%，其中出口增长6%；城市居民人均可支配收入增长8%，农民人均纯收入增长7%；城镇登记失业率控制在3.5%以内；人口自然增长率控制在2.2%。以内；万元生产总值能耗下降5.1%，SO2和COD排放分别减少2%和6%。

(一)落实宏观调控政策，保持经济平稳较快增长。

着力增强投资拉动力。紧紧抓住国家扩大内需的政策机遇，加大固定资产投入，增强投资对经济发展的拉动作用。继续开展项目推进年活动，加快建设一批先进装备制造业、高新技术产业、现代服务业等重大产业项目，交通、能源、水利等重大基础设施项目，旧城旧村改造、保障性住房建设等民生项目，集中抓好年度投资562亿元的148个市级重点项目。充分发挥国家投资的导向作用，最大限度地激活社会投资，积极培育新的投资主体。进一步优化投资结构，严把项目准入关，引导投资向民生保障、“三农”、基础设施、节能环保、自主创新等重点领域倾斜，坚决防止高耗能、高污染、低水平重复建设，以投资结构的持续优化带动经济结构的不断升级。

努力扩大消费需求。调整收入分配格局，提高农民和城市低收入者的收入水平，提高居民消费能力。完善鼓励消费的各项政策措施，健全城乡流通网络，进一步改善消费环境。加大农村市场开拓力度，继续抓好“万村千乡”市场工程和“家电下乡”工作，引导城镇商业网点向农村延伸，促进耐用消费品更多地进入农村家庭。年内新建改造标准化农家店1000个，销售“家电下乡”产品10万件。积极培育消费热点，开发满足不同层次消费需求的商品和服务,鼓励汽车消费，促进节假日和会展消费，扩大文化、旅游、健身、休闲消费，推动房地产业平稳发展。

保障企业健康发展。充分估计国际金融危机对实体经济的严重冲击，千方百计帮助企业应对危机、渡过难关。用足用活各级鼓励企业发展的优惠政策，帮助解决实际困难，增强企业发展信心。引导企业增加研发和创新投入，及时调整产品结构，坚持让利不让市场，保住宝贵的市场份额。鼓励有实力的企业兼并收购落后困难企业，鼓励优势企业强强联合，鼓励关联产业、上下游企业联合重组。规范企业内部管理，引导企业练内功、挖潜力、降成本、增效益。开展行政事业性收费专项治理，从今年1月1日起，取消190项收费项目，将19项收费标准降低40%，其他行政事业性收费均降低10%，全年减轻企业负担4.6亿元。优化金融生态环境，支持金融机构扩大信贷投放，加大对重点产业、重点企业和中小企业的资金倾斜力度。大力推进企业境内外上市，加快设立创业投资引导基金，增强担保机构融资能力，搞好村镇银行和小额贷款公司试点。争取新增建设用地指标，盘活存量土地，缓解用地紧张的矛盾。

(二)积极调整经济结构，走高端化、特色化、集约化发展道路。

膨胀发展先进制造业。加快工业信息化步伐，坚持用高新技术改造传统产业，着力提高制造业技术装备水平，推动产业、企业和产品向高端化进军。巩固提升汽车、电脑、手机、葡萄酒、黄金等优势特色产业，快速膨胀核电、船舶、现代化工、新型冶金等新兴产业。年内，全市规模以上工业主营业务收入达到8800亿元，增长12%。新上和建设一批带动产业升级的重大项目。启动和建设万华石化产业园、冰轮工业园，推动工业企业“退城进园”。加快海阳核电项目建设，年内投资51亿元完成核岛、常规岛负挖及部分配套设施建设。抓好船舶产业聚集区和大宇造船、中柏京鲁船业、莱佛士海洋重工等项目建设。加大对宝钢精密钢管和东方不锈钢等新型冶金项目的扶持力度。实施名企名牌战略，继续推进“3·50工程”，培强做大骨干企业，加大品牌建设力度，争取再创3件中国驰名商标。

突破发展现代服务业。优化提升商贸餐饮业。积极培育城市商业中心和特色街区，重点抓好三站市场新区等市区十大商贸设施项目建设，做大流通品牌和龙头企业。加快发展旅游会展业。进一步做优做精中心区和“蓬长龙”“1＋3”旅游板块，重点推进总投资46亿元的大南山生态景区建设，抓好塔山娱乐园扩建和昆嵛山森林生态区等旅游开发项目，打造旅游新亮点。整合旅游资源，培育大型旅游龙头企业，开拓国内外多层次市场。办好第三届烟台国际葡萄酒节、中国北方旅游交易会等各类展会。快速膨胀金融商务服务业。培育壮大恒丰银行、市商业银行等金融机构，积极引进内外资银行和保险机构，加快建设烟台金融中心。利用良好的制造业发展基础和交通设施条件，大力发展现代物流等生产性服务业，集中培育六大物流园区。启动建设烟台软件园和服务外包基地，大力发展软件、动漫、设计等高端服务业。争取全年服务业增加值达到1190亿元，增长12%。

培植区域特色经济。加快发展海洋经济和临港产业。有序开发海洋资源，提高海水养殖、水产品加工和远洋捕捞能力，积极培育海洋生物、海水综合利用、海洋能源等新兴产业。年内力争海洋产业产值增长15%以上。合理布局临港产业带，加快发展石化、冶金、船舶、电子信息等临港工业。积极发展港口物流，建设芝罘湾港区大型集装箱中转站，支持烟台港与中国神华、宝钢集团开展战略合作，促进航线、航班和运量互动增长。放手发展民营经济。深入实施千家小企业培育、中小企业成长和特色产业提升计划，扶持发展特色产业集群。完善公共服务体系，优化中小企业发展环境，力争全年民营经济增加值和上缴税金均增长12%以上。统筹发展县域经济。鼓励各县市区立足自身优势培育特色经济，依靠大企业、大项目带动产业发展，推动经济强县在全国百强、全省30强中进档升级，加大帮扶栖霞力度，支持长岛科学发展。

加快自主创新步伐。高度重视科技进步在应对危机、促进增长中的关键性作用，以建设创新型城市为目标，大力发展高新技术产业，积极培育先进制造、新材料、电子信息、生物制药等重点行业，集中扶持氨纶集团等十个具有规模效应、先声麦得津等十个成长性较强的高新技术企业，抓好荣昌“泰爱”等十个自主创新项目。全年高新技术产业产值达到3300亿元以上，占规模以上工业产值的比重达到40%以上。加快烟台高新区规划建设，打造新的高新技术产业隆起带、产学研结合示范区和高端服务业聚集区。完善科技创新体系，突出企业在自主创新中的主体地位，充分发挥驻烟高校、科研院所的作用，积极推进中科院烟台海岸带研究所、中科院烟台综合技术转化中心和留学人员创业园区建设。

加大节能减排力度。全面加强节能、节水、节地、节材和资源综合利用工作。以电力、水泥行业为重点，严格执行差别电价、超标耗能加价等政策，淘汰落后产能，争取关停11万千瓦小火电机组，关闭13条水泥立窑生产线。继续抓好110家重点用能企业节能管理，培育10个循环经济产业链条。加快建设治污减排重点工程，新建扩建套子湾污水处理厂二期、南郊污水处理厂和龙口黄水河污水处理厂，升级改造莱州市污水处理厂，建设改造9个无害化垃圾处理项目。

(三)纵深推进改革开放，增强发展的活力与动力。

拓展开放领域。准确把握金融危机引发的国际产业资本流动的新趋势和国际市场格局调整的新变化，采取更加灵活的开放措施，推动开放型经济转型升级。提高利用外资水平。精心论证策划一批大项目、好项目对外招商，突出抓好重大先进制造业、高端服务业、高新技术、总部经济、基础设施等领域的招商引资，推动现有外资企业增资扩股，吸引国外大企业跨国并购我市企业。跟踪抓好在谈、在批、在建项目，办好重大招商活动，完善招商项目推进机制。积极调整外贸策略。转变外贸发展方式，走以质取胜、集约化、多元化的发展路子，巩固传统市场，扩大欧美等高价位市场，开发受金融危机影响较小的新兴市场，扩大具有自主知识产权和自主品牌的产品出口，促进加工贸易转型升级，鼓励先进技术、关键设备和重要能源原材料进口。年内高新技术产品和机电产品出口比重分别达到35%和65%以上。全面优化开放环境。保护外资企业合法权益，强化部门联动服务机制，排除影响发展的制约和障碍。帮助企业开拓国际市场和化解贸易磨擦，完善大通关体系，进一步降低通关成本，确保出口退税“应退尽退”。加强开放载体建设。推动烟台经济技术开发区在更高层次上创新发展，争取国家批准设立烟台保税港区，发挥台湾农民创业园政策效应，推进省级园区再上新水平。加快实施“走出去”战略。抓好境外资源的开发利用，积极发展境外农业，支持骨干企业建立境外生产基地。鼓励企业开展对外承包工程和劳务合作。加强对外经济文化交流与合作，提高烟台的国际知名度和影响力。

深化各项改革。坚持社会主义市场经济改革方向，突出重点领域和关键环节，积极采取有利于保增长、扩内需、调结构的改革措施。大力推进农村综合改革。稳定和完善农村基本经营制度，规范农村土地管理体制改革，加强土地承包经营权流转管理和服务，扎实推进集体建设用地依法有序流转，扶持发展农民专业合作社。加大企业改革力度。加快国有经济战略布局和结构调整，完善国有资产管理体制和监管方式，建立更加规范的现代企业制度。深入推进财税体制改革。全面落实增值税转型改革，抓好部门预算、国库集中支付、政府采购、投资评审和收支两条线改革。加强财源建设，强化税收征管，增强各级财政的持续增收能力和保障能力。深化医药卫生体制改革，为人民群众提供安全、有效、方便、价廉的医疗卫生服务。推动社会保障、文化教育和事业单位等各项改革，完善保障科学发展的体制机制。

(四)打造中心城市优势板块，提升对区域发展的辐射带动力。

加快五区融合步伐。按照建设大城市、大市区的要求，调整完善中心城市发展规划，以大南山、夹河、辛安河“一山两河”和海岸线、芝罘岛、崆峒岛、养马岛“一线三岛”的建设为纽带，带动五区结合部和城乡连接点的开发改造，着力打造高档次旅游景观带、高品位居民商住区和高层次产业聚集区。高标准推进芝罘南部、莱山中部、牟平北部区域的开发建设，膨胀中心城市规模。规划启动福山南部、开发区西部区域的景观改造，提升城市门户形象。搞好重点区域、重点地段的城市设计和精品建设，启用新火车站、文化中心等标志性建筑，加快北马路周边、毓璜顶医院周边、胜利小区、幸福区片等区域开发改造。调整市区管理权限，理顺管理体制，统筹路网、公交、给排水等市政设施建设。加快发展城市经济，优化五区产业布局，增强综合发展实力。

完善基础设施体系。全面构建“两纵一横”铁路网络。完成蓝烟铁路电气化改造主体工程，开工建设龙烟铁路和青烟威荣城际铁路。三条铁路改造建成后，全市干线铁路里程达到470公里以上，货物运输能力达到1亿吨，年发送旅客6000万人次，均比2008年提高1倍以上。提高渤海铁路轮渡综合物流水平，积极争取中韩铁路轮渡项目。构筑完善的道路交通网络。加快建设烟台港西港区、龙口港区、莱州港区疏港高速公路和海阳跨海大桥，开工建设烟台至海阳高速公路、同三高速福山立交、绕城高速莱山立交等工程。启动红旗中路财校段、凤凰南路等16条城区道路工程。高效推进港口建设。芝罘湾港区三突堤集装箱码头投入使用,完成西港区通用泊位主体工程。推进西港区30万吨级原油码头项目和输油管线项目、龙口港区煤炭专用泊位项目。争取开工建设总投资45亿元的烟台潮水国际机场。

提高城市管理水平。加强城市数字化管理，完善信息平台系统，扩大数字化管理覆盖范围。推进城市社区建设，按照人性化、规范化、生态化的要求，打造一批体现城市管理水平和文明程度的样板社区。健全城市交通体系，坚持“公交优先”，完善烟台市公共交通规划，强化牟平区、福山区和新建居民小区公交配套。加快城市公益设施建设，年内市区新增集中供热面积120万平方米、管道燃气用户1万户。搞好市区环境综合整治，逐步清理违章建筑，杜绝乱搭乱建现象。搞好城市经营，更加注重塑造城市品牌，挖掘自然人文、商业品牌、城市名片等无形资产的潜力，使城市发展更具内涵、更有底蕴。

优化城市生态环境。以生态市建设为总抓手，集中实施蓝天、碧水、青山、绿地“四大生态工程”。蓝天工程，加大汽车尾气、工业废气、扬尘污染等治理力度，建立环境应急预警系统，加强重点污染源环境安全监管。碧水工程，全面实施门楼水库水源地治理，对夹河、黄水河、鱼鸟河等河道进行水环境综合治理，积极开展农村河溪池塘清洁整治和生态小流域建设。实施海洋生态修复，控制和治理污水排放，推进莱州湾万亩海上蓝色牧场建设。青山工程，继续实施“三年大造林”工程，重点搞好山区和海防林绿化，年内完成造林绿化19.6万亩。绿地工程，提高城市道路、广场、公园等区域绿化水平，新建逛荡河、凤凰湖等城市公园，建设改造10处社区公园。

(五)加快建设社会主义新农村，构筑城乡一体化发展新格局。

大力发展现代农业。坚持把粮食生产放在现代农业的首位，坚守耕地面积、粮田面积和粮食总产“三条底线”，确保粮食安全。发展品牌农业，推进新一轮农业结构调整，开发特色果蔬产品，提升畜牧业发展水平，抓好农业标准化生产。年内新增水果种植面积20万亩以上，新建改建各类畜禽标准化饲养场1000个，新发展农业标准化生产基地50万亩。加快农业产业化进程，新增销售收入过亿元的农业龙头企业6家。加强农技推广体系建设，着力发展大型先进农机具，提高农业现代化水平。

改善农村生产生活条件。加强农村水利基础设施建设，完成3座中型水库除险加固主体工程和209座小型水库除险加固任务。推广农业高效节水技术，建好王屋水库大型灌区配套工程，年内发展节水灌溉面积18万亩。继续实施农村饮水安全工程，改造惠及400个村、36万人的自来水设施。搞好农村公路建设，改造农村公路400公里。完成20个乡镇卫生院、500个村卫生室标准化改造，全市80%的村建成不低于100平方米的文化大院或文化活动室，40%的村建成达标的农家书屋。建设一批农村沼气工程，新增沼气用户3万户。加大小城镇和农村社区建设力度，开展村庄综合整治活动，推行生活垃圾集中处理。破解城乡二元结构，按照城市形态要求建设农村基础设施和公共服务设施，逐步缩小城乡差距，实现城乡一体化发展。

落实强农惠农政策。增加“三农”投入，保证各级财政农业投入增幅高于经常性收入增幅，提高土地出让收益和新增耕地占用税用于农业的比例，鼓励发展农村二、三产业。全面落实粮食最低收购价格政策，扩大农资综合直补、良种补贴、农机具购置补贴范围。

(六)全面发展社会事业，创建和谐稳定模范城市。

推动文化大发展大繁荣。深入挖掘文化资源，精心策划葡萄酒文化、开埠文化、道教文化、胶东民俗风情文化等特色文化品牌，把国际葡萄酒城的品牌打造成烟台的地理商标。弘扬烟台精神，繁荣文艺创作，继承和发扬传统文化艺术。引进先进经营理念和运行机制，高水平策划一批特色剧目和演艺活动，丰富群众文化生活，塑造新的城市文化品牌。争创1个国家级、3-5个省级文化产业示范基地。鼓励新闻出版、传媒展览等文化产业繁荣发展，支持文化龙头企业做大做强。完成中心区有线电视数字化整体转换。深入开展文明创建活动，展现全国文明城市风采。

促进社会事业全面进步。坚持教育优先原则，扎实推进素质教育，大力发展职业教育，支持驻烟高校发展，鼓励新建大学项目。健全完善义务教育经费保障机制，按照高于一般建筑防震等级要求加固中小学校舍，实施中小学办学条件标准化工程和农村中小学“两热一暖一改”工程。提高农村中小学公用经费基本标准，继续对家庭经济困难学生给予生活补助，不让一个学生因贫失学。加大人才引进与培养力度，建立与科学发展、产业需求相适应的人才队伍。搞好城乡公共体育设施建设，广泛开展全民健身活动，不断提高竞技体育水平。做好第三届亚洲沙滩运动会场馆建设及各项筹备工作，承办好第十一届全运会烟台赛区比赛。健全公共卫生服务体系，抓好专科医院建设，扶持中医药事业发展。完善城市社区卫生服务和农村三级医疗卫生服务体系，用3年时间使全市村卫生室均达到省要求标准。加强人口与计划生育工作，继续稳定低生育水平。强化气象和防震工作，提高防御自然灾害的能力。卓有成效地做好支援地震灾区恢复重建工作。抓好外事侨务、史志档案、人民防空、民族宗教、仲裁、红十字会以及国防教育、国防动员、民兵预备役、双拥共建等工作。

推动社会和谐稳定。以创建和谐稳定模范城市为目标，深入开展五五普法教育，加强社会治安综合治理，严厉打击各类违法犯罪活动。强化应急管理，加强预案预警、应急救援和应急保障体系建设，增强应对突发事件的能力。加大安全生产监管、执法检查和综合整治力度，开展“安全生产基石工程年”活动，减少和遏制各类事故发生。继续抓好信访工作，维护群众合法权益，最大限度减少不和谐因素、增加和谐因素。

(七)切实保障和改善民生，提高群众满意度和幸福感。

千方百计扩大就业。实施更加积极的就业政策，支持创业带动就业，鼓励自主创业、自谋职业。抓好大中专毕业生就业工作，探索建立大学生创业孵化基地。拓宽农民工就业渠道，城乡基础设施建设和新增公益性就业岗位尽量向农民工倾斜，支持农民工返乡创业。加大对下岗人员和农民工的免费培训力度，支持就业容量大的劳动密集型企业发展。强化失业调控，按照《劳动合同法》规范企业用工行为，引导企业不裁员或少裁员。抓好困难群体就业帮扶工作，确保城镇零就业家庭和农村零转移就业贫困家庭实现“动态消零”。加大就业资金投入，加强街道、乡镇公共就业服务体系建设，确保各项促进就业的扶持政策落实到位。全市新增城镇就业11万人以上、农村劳动力转移就业2万人以上。

健全社会保障机制。扩大社会保险覆盖范围，提高企业退休人员养老待遇、失业职工失业待遇。推进城镇居民基本医疗保险制度改革，将驻烟高校大学生纳入保险范围，城镇居民参保率达到80%以上。适当提高医疗保险待遇支付标准，调整完善职工工伤、生育保险政策。推行和完善新型农村养老保险制度与合作医疗制度，提高参合农民受益幅度。加强社保基金管理，规范基金管理流程。

搞好弱势群体救助。扩大城乡居民最低生活保障覆盖面，争取城镇低保覆盖率达到1.5%以上，农村低保覆盖率达到3.5%以上，农村低保标准每人增加100元。完善社会救助体系，加强法律援助工作，大力发展慈善事业，继续开展爱心捐助活动。重视发展老龄事业，切实保障妇女和未成年人权益，关心和支持残疾人事业。抓好市老年福利服务中心二期、流浪未成年人救助保护中心和儿童福利院等工程建设。完成400户农村贫困残疾人危房改造，巩固建设100处残疾人就业扶贫基地、100个“老年之家”示范点。

提高住房保障水平。推进保障性安居工程，加大廉租住房保障力度，加快经济适用房建设步伐，探索建立政府限价房、农民工住房等制度，多渠道解决城市中低收入群众住房困难。年内全市新建经济适用房5000套。采取货币补贴与实物配租相结合的方式，对低收入家庭实现廉租住房“应保尽保”。

保持物价基本稳定。抓好地方储备粮等重点物资储备管理，加强市场价格监管，整顿市场价格秩序，完善价格调节基金制度，健全物价上涨与提高低收入群体生活保障标准联动机制。居民消费价格涨幅控制在4%以内。

保障食品药品安全。深入开展食品安全示范创建活动，建立食品安全风险调查评估机制，提高食品药品安全水平。全面推行农产品质量例行监测制度，加强农产品、畜产品、水产品质量检测和海洋环境监测等设施建设，强化出口食品、农产品质量安全区域化管理。健全卫生监督体系，抓好餐饮卫生、职业卫生、学校卫生、公共场所卫生监督管理，让群众吃得放心，用得安心。

各位代表！保障和改善民生，是各级政府义不容辞的职责。当前，国内外经济形势严峻，必然给民生工作带来许多困难。越是在困难的时候，越是要高度关注民生。我们将始终牢记为人民服务的宗旨，加大民生投入，改善群众生活，保障群众利益，努力开创全市人民安居乐业、共享和谐的新局面！

三、全面加强政府自身建设

新的形势下，政府工作任务更加艰巨。全市政府系统必须强化忧患意识、树立世界眼光，改进管理，优化服务，提高效率，不断增强凝聚力、创造力和战斗力，切实肩负起推动经济社会又好又快发展的历史重任。

(一)科学把握形势，强化政府调控职能。深入开展学习实践科学发展观活动，领会和把握科学发展观的深刻内涵，学习新知识，研究新情况，解决新问题，提高科学判断形势的能力、驾驭市场经济的能力和应对复杂局面的能力，把握发展机遇，破解发展难题，培育发展优势。面对当前严峻的经济形势，善于激发和调动广大干部群众想发展、谋发展的积极性，营造奋发进取、干事创业的浓厚氛围。正确处理当前与长远的关系，坚持一切从实际出发，强化对经济运行的调控，真正把保增长、促发展的任务落到实处。按照社会主义市场经济体制的要求，全面履行经济调节、市场监管、社会管理和公共服务职能，努力做到权责对等、分工合理、执行顺畅，不断提高政府的执政能力和水平。

(二)推进依法行政，全面规范行政行为。认真执行国务院、省政府关于加强市县政府依法行政的有关规定，严格依法办事，认真履行职责，科学民主决策。完善重大事项公示制度和听证制度，保证人民群众依法行使知情权、参与权和监督权。加大行政综合执法改革力度，进一步明确行政执法权限，减少执法层级，规范执法程序。严格实行行政执法责任制和执法过错追究制，切实做到有法必依、违法必究，公正执法、文明执法。自觉接受市人大及其常委会的法律监督、工作监督和市政协的民主监督，自觉接受群众监督和舆论监督，高度重视人大代表建议和政协提案的办理工作，广开参政议政渠道。

(三)创新管理方式，不断提高工作效能。推进行政管理体制改革，改进政府管理方式和工作手段，提高办事效率和服务水平。进一步优化发展环境，注重运用经济、法律手段管理经济社会事务，加强规划引领、政策指导、信息服务等工作，营造激发市场主体活力的制度平台。建立政府主导、社会参与的公共服务机制，不断提高服务能力。深化行政审批制度改革，深入实施“阳光政务”工程，积极推行政务公开，抓好电子政务建设。落实耕地保护、节能减排、安全生产、食品安全等各项责任，建立健全科学发展、和谐发展的保障机制。

(四)坚持以人为本，切实做到执政为民。大力弘扬埋头苦干的作风，深入开展调查研究，把以人为本的执政理念体现在各项工作部署中，落实到经济社会发展的各个方面。尊重群众的主体地位，发挥群众的首创精神，坚持问政于民、问需于民、问计于民，牢固树立服务意识，扎扎实实为基层、企业和群众办实事、解难题。充分发挥市长公开电话和市长信箱作用，了解群众愿望，倾听群众呼声，关注群众疾苦。继续实施为民服务实事工程，解决好人民群众最关心、最直接、最现实的利益问题。

(五)加强廉政建设，努力维护政府形象。坚持标本兼治、综合治理、惩防并举、注重预防的方针，加强惩治和预防腐败体系建设。坚决贯彻执行中央、省、市关于廉洁自律的各项规定，认真落实党风廉政建设责任制，加强对领导干部的廉政教育，推进廉政和反腐败制度建设，严肃查处违法违纪案件，坚决纠正损害群众利益的不正之风。加大行政监察力度，强化对建设工程、土地出让、产权交易和政府采购等领域的监管，形成公开、公平、公正的运行机制。始终保持艰苦奋斗、勤俭节约的优良传统，坚决取消不合理、不必要的行政开支，降低行政成本。加强公务员能力和作风建设，努力打造一支人民满意的公务员队伍。

各位代表！烟台的发展已经进入一个关键时期。我们既要在各种风险和挑战面前攻坚克难、开拓局面，又要为全市经济社会发展跃上新台阶积蓄力量、打牢基础。任务艰巨，使命崇高！让我们更加紧密地团结在以胡锦涛同志为总书记的党中央周围，高举中国特色社会主义伟大旗帜，以邓小平理论和“三个代表”重要思想为指导，深入贯彻落实科学发展观，在中共烟台市委的正确领导下，团结和依靠全市广大干部群众，振奋精神、坚定信心，抢抓机遇、开拓进取，为夺取改革开放和现代化建设的新胜利而努力奋斗！
